# Clinical Efficacy of Laparoscopic Orchiopexy With the Modified Prentiss Maneuver for Non-palpable Testis Near the Internal Ring

**DOI:** 10.3389/fped.2022.906739

**Published:** 2022-05-27

**Authors:** Tian-Qu He, Fang-Yun Tong, Zhi Wang, Yu Liu, Jian-Jun Hu, Yi-Fu Chen, Lei Tu, Jun He, Yao-Wang Zhao

**Affiliations:** Department of Urology, Hunan Children's Hospital, Changsha, China

**Keywords:** laparoscopic, orchiopexy, modified Prentiss maneuver, non-palpable testis, cryptorchidism

## Abstract

**Objective:**

To compare the clinical efficacy and safety of laparoscopic orchiopexy with the modified Prentiss maneuver (LOMPM) and laparoscopic trans-inguinal orchiopexy (LTIO) for the treatment of non-palpable testis (NPT) <1 cm from the internal ring.

**Methods:**

Children with unilateral NPT who underwent laparoscopic orchiopexy at our center between February 2018 and January 2021 were retrospectively analyzed. According to the surgical method, they were divided into LOMPM and LTIO groups. The operation time, postoperative pain degree, postoperative complications and follow-up results were compared between the two groups.

**Results:**

A total of 98 patients were included in this study, including 41 cases in the LOMPM group and 57 cases in the LTIO group. All patients underwent successful surgery. The LOMPM group was superior to the LTIO group in terms of postoperative testicular position (lower scrotm: 90.2 vs. 71.9%, *P* = 0.026). There were no significant differences in operation time, postoperative pain score, and complications between the two groups. Preoperative testicular volume, postoperative testicular volume, and testicular growth rate in the LOMPM group were comparable to those in the LTIO group. There were no testicular atrophy, inguinal hernia and hydrocele in both groups after operation.

**Conclusions:**

LOMPM was comparable in safety to LTIO, but LOMPM had a good post-operative testicular position, and was suitable for the treatment of NPT near the internal ring.

## Introduction

Undescended testis (UDT) is a relatively common reproductive system disease in children. The prevalence of UDT is about 1–4.6% in normal-weight male neonates, and is as high as 20–30% in preterm infants or neonates weighing <2500 (1, 2). UDT reduces long-term fertility potential, and is a risk factor for testicular malignancy and testicular torsion (1, 2). Orchidopexy is the main method for the treatment of UDT. For non-palpable testis (NPT), traditional inguinal incision surgery is blind, and the difficulty of exposure leads to limited length of free spermatic cord, and the position of testis descending is usually unsatisfactory (3–6). In 1955, Prentiss et al. (7) first proposed two anatomical concepts, the spermatic surgical triangles and the lateral spermatic ligament, and applied them to orchidopexy. This technique shortens the descent of the testis by dividing the transverse abdominal fascia, inferior epigastric vessels, and lateral spermatic ligament, and performing retroperitoneal dissection to offset the spermatic vessels medially (7, 8), which is considered an effective and necessary surgical technique for the treatment of high UDT. Subsequently, Ayub et al. (9) described a modified method: a small incision was made in the transverse fascia above the pubic tubercle for the extraction of the testis, thus avoiding segmentation and ligation of the inferior epigastric vessels.

In recent years, laparoscopy has become the standard procedure for the diagnosis and treatment of NPT (2, 6). Laparoscopy can not only determine the location and size of the testis, but also maximize the mobilization of the spermatic cord in the abdominal cavity (3, 10). At the same time, the laparoscopic technique can directly establish the abdominal cavity-scrotal channel, so that the modified Prentiss maneuver has been widely used. In addition, descending the testis through the inguinal canal restores the normal path of testicular descent, which also provides an anatomical basis for sparing the gubernaculum. Recent findings suggest that descending the testis through the internal ring during staged laparoscopic Fowler-Stephens orchiopexy can result in a lower probability of testicular atrophy and a better temperature for testicular development (11–13). To our knowledge, there is no literature comparing the clinical efficacy of two different descending channels in vessel-intact laparoscopic orchiopexy. We hypothesized that the modified Prentiss maneuver would be as safe as trans-inguinal orchiopexy, and achieve a better postoperative position without affecting testicular development.

This study retrospectively analyzed children with unilateral NPT who underwent laparoscopic orchiopexy at our center between February 2018 and January 2021. The purpose of this study was to compare the efficacy of laparoscopic orchiopexy with the modified Prentiss maneuver (LOMPM) and laparoscopic trans-inguinal orchiopexy (LTIO) in the treatment of NPT with <1 cm from the internal ring.

## Materials and Methods

This study was conducted in accordance with the declaration of Helsinki. This study was conducted with approval from the Ethics Committee of Hunan Children's Hospital. Written informed consent was obtained from the guardians of the participants.

### Patients

Children with unilateral NPT who underwent laparoscopic orchiopexy between February 2018 and January 2021 in our center were retrospectively analyzed. All operations in this study were performed by two surgeons. Since our center only started performing laparoscopic orchiopexy with the modified Prentiss maneuver in August 2019, two cohorts could be formed. According to the difference of surgical methods, they were divided into LOMPM group (August 2019-January 2021) and LTIO group (February 2018–July 2019).

#### Inclusion Criteria

(1) The testis was of sufficient size confirmed by diagnostic laparoscopy; (2) The testis was located in the inguinal canal or the abdominal cavity <1 cm from the internal ring. Exclusion criteria: history of hormone therapy, peeping testis/inconsistently palpable cryptorchidism, cryptorchidism-related syndrome, or incomplete follow-up data.

### Clinical Data

Demographic data included age at surgery, height, BMI, laterality, preoperative hernia or hydrocele, and testicular volume by color Doppler ultrasound. Perioperative data were collected on surgical method, operative time, whether the internal ring was closed, UDT location (intra-abdominal or inguinal canal), postoperative face, legs, activity, crying, consolability (FLACC) pain sore and complications, etc.

All patients underwent regular outpatient follow-up at postoperative 1, 3, and 6 moths, and yearly. The final position of the testis was confirmed by clinical examination, and color Doppler ultrasonography was performed at postoperative 1, and 6 months, and annually to check testicular size and blood flow.

The main evaluation criteria for this study were the location and viability of the testis after surgery. The location of the testis after surgery is divided into the lower scrotum (the testicles being located in the lowest position of the scrotum), the middle scrotum (testicles not being located in the lowest position of the scrotum, but still inside the scrotum), and the neck of the scrotum and above (the testicles being located in the groin area). Testicular retraction is defined as the testis located at and above the neck of the scrotum (2, 13). Testicular volume was calculated using the ellipse formula = length × width × height × 0.523 (2, 14), and the values were from the results of ultrasonography. Testicular growth rate is the ratio of postoperative testicular volume to preoperative testicular volume. Testicular atrophy is defined as a testicular growth rate of <0.5, that is, a loss of more than 50 percent of the postoperative testicular volume relative to the preoperative (14). The testicular position and volume of the two groups were compared by outpatient follow-up 1 year after surgery.

### Operative Technique

#### Laparoscopic Orchiopexy With the Modified Prentiss Maneuver

**Figure 1:**
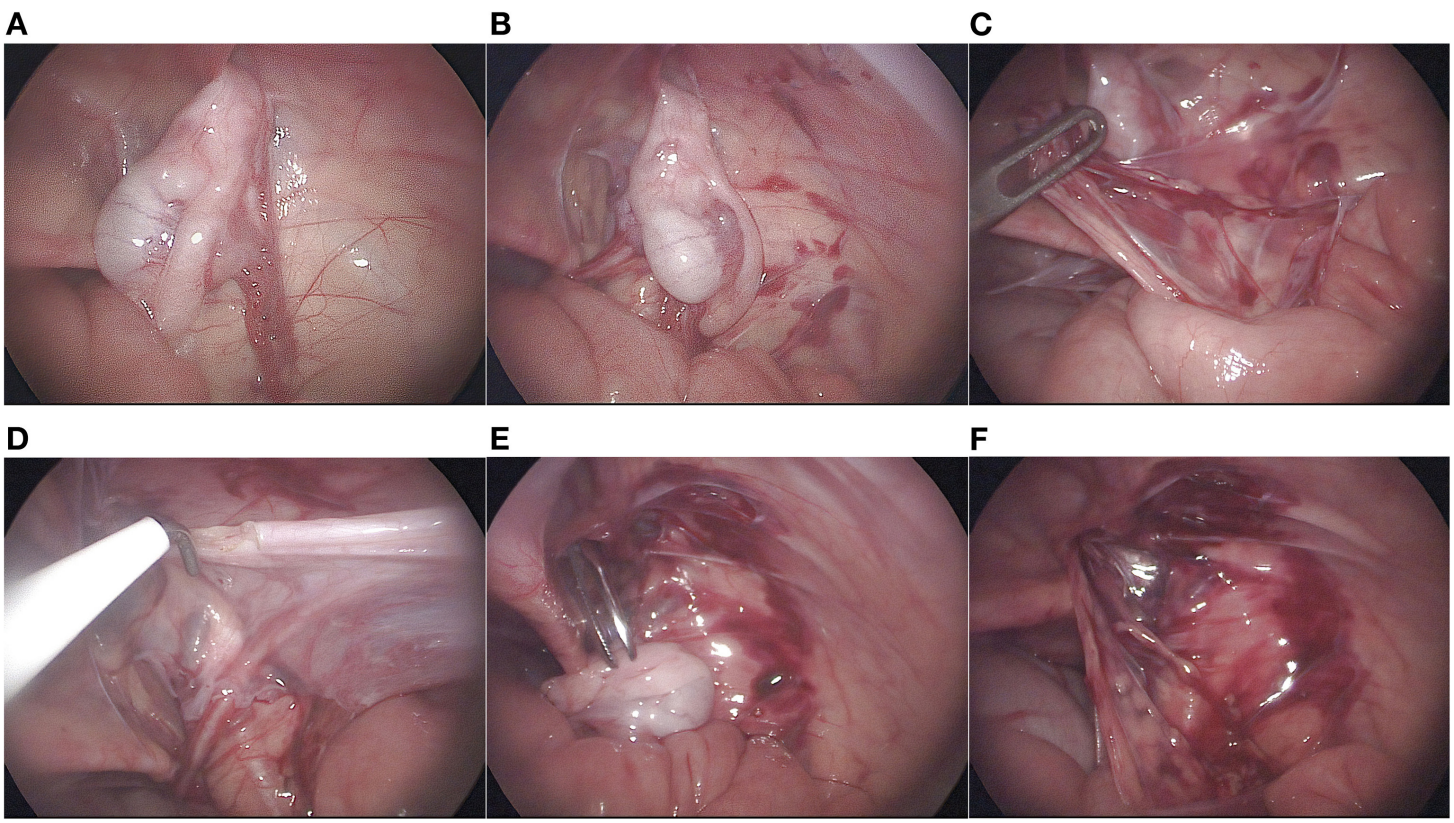
Surgical procedure. **(A)** the position of the testis and whether the internal inguinal ring is patented were observed; **(B)** incision of the peritoneum lateral to the spermatic vessels and medial to the vas deferens; **(C)** Mobilization of spermatic vessels proximally; **(D)** gubernaculum was transected; **(E)** Establishment and expansion of the medial channel of the inferior epigastric vessels; **(F)** Descending testis under laparoscopic direct vision.

**Figure 2:**
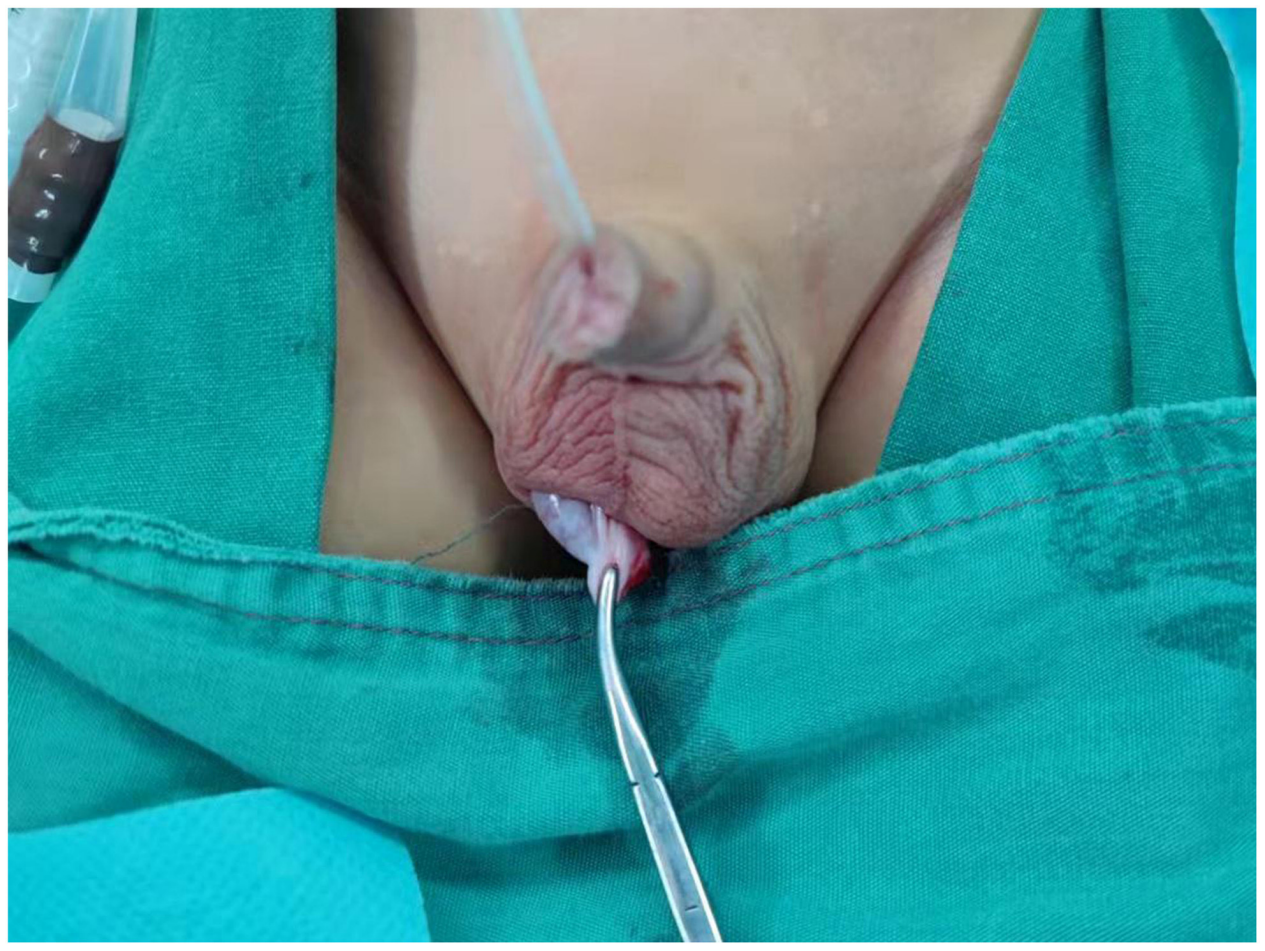
The testis descended into the scrotum.

After general anesthesia combined with epidural anesthesia, the patient was placed in a supine position. We routinely disinfected and draped the surgical area, inserted a urinary catheter, and emptied the bladder. A 5 mm Torcar was placed in the umbilicus using the open Hasson's technique to establish a viewing channel. A pneumoperitoneum (pressure 8–10 mmHg) was established. A 5 mm Trocar was placed on both sides of the midclavicular line below the umbilicus to establish an operating channel. The condition of the internal ring and the position of the testis were observed (Figure 1A). The peritoneum lateral to the spermatic vessels and medial to the vas deferens was incised, leaving the distal triangle of the peritoneum between the spermatic cord and the vas deferens (Figure 1B). The peritoneum on the surface of the spermatic cord was incised, and the spermatic cord were sufficiently dissociated to the lower pole of the kidney to mobilize the testis (Figure 1C). When identifying, grasping, and transecting the gubernaculum, attentions to the vas deferens were paid to avoid damage to the looping vas deferens (Figure 1D). The testis was placed over the iliac vessels. A transverse incision was made in the scrotum, a subdartos pouch was made, the sarcophagus was cut, and the hemostatic forceps were passed from the scrotal incision through the transverse abdominal fascia into the medial side of the inferior epigastric vessels. The descending channel of the testis was gradually dilated by opening and closing the hemostatic forceps to ensure smooth passage of the testis (Figure 1E). The stump of the gubernaculum was clamped, and the testis was lowered into the scrotum under direct vision of the laparoscope (Figure 1F). A retractor was used in the scrotal incision to expose the external inguinal ring. The spermatic cord were inspected for torsion, and the testis was secured into the subdartos pouch (Figure 2).

#### Laparoscopic Trans-inguinal Orchiopexy

Anesthesia method, trocar location, and the methods of testis mobilization and testicular fixation were similar to LOMPM. After mobilizing the testis, the laparoscopic grasping forceps was pushed into the internal ring to reach the outside of the scrotum, then the hemostatic forceps were guided into the abdominal cavity, and finally the testis was grabbed and pulled it down to the scrotum.

Both surgical approaches did not close the internal ring peritoneum.

### Statistical Analysis

The statistical software of SPSS 22.0 was used. The measurement data were expressed as mean ± standard deviation (SD), and the independent samples *t* test was used for the comparison between groups. The count data was expressed by *n* (%), and the chi-square test was used for the comparison between groups. *P* < 0.05 indicated a statistically significant difference.

## Results

A total of 98 patients were included in this study, including 41 cases in the LOMPM group and 57 cases in the LTIO group. The basic conditions and clinical characteristics of all patients were shown in Table 1. There were no significant differences in age, height, BMI, laterality, hernia or hydrocele between the two groups. There were also no significant differences in the initial testis location (intra-abdominal or inguinal tubule) and the processus vaginalis (open or closed) between the two groups.

**Table 1:** Comparison of preoperative, and intraoperative data between the two groups.

	**LOMPM group**	**LTIO group**	***p*-value**
Number	41	57	
Age (months)	26.85 ± 18.46	26.02 ± 20.00	0.834
Height (cm)	86.37 ± 12.34	86.14 ± 12.82	0.931
BMI	16.40 ± 1.69	16.57 ± 1.66	0.622
Laterality, *N* (%)			0.683
Light	17 (41.5)	26 (45.6)	
Right	24 (58.5)	31 (54.4)	
Hernia or hydrocele, *N* (%)	8 (19.5)	11 (19.3)	0.979
Initial location, *N* (%)			0.897
Intracanalicular	29 (70.7)	41 (71.9)	
Abdominal	12 (29.3)	16 (28.1)	
Processus vaginalis, *N* (%)			0.695
Open	33 (80.5)	44 (77.2)	
Closed	8 (19.5)	13 (22.8)	

All patients underwent successful surgery without additional inguinal incisions. There were no significant differences in operation time and postoperative pain scores between the two groups. No patient used pain medication. One patient (1.8%) in the LTIO group developed poor wound healing of the scrotum after surgery, and two-stage suturing was performed. Postoperative scrotal hematoma occurred in 5 patients (12.2%) in the LOMPM group and in 6 patients (10.5%) in the LTIO group, with no significant difference. Hematomas were confined to the scrotum and healed without special intervention. No incision infection and abdominal incisional hernia occurred in all cases (Table 2).

**Table 2:** Comparison of operation time, postoperative data between the two groups.

	**LOMPM group**	**LTIO group**	***p-*value**
Number	41	57	
Operation time, min	41.32 ± 5.8	42.84 ± 5.65	0.196
Postoperative pain score (24 h)	1.24 ± 1.16	1.18 ± 1.18	0.776
Postoperative pain score (48 h)	0.39 ± 0.63	0.39 ± 0.65	0.974
Testicular retraction, *N* (%)	1 (2.4)	2 (3.5)	1.000
**Testicular position**		
Lower scrotm, *N* (%)	37 (90.2)	41 (71.9)	0.026
Mid scrotm, *N* (%)	3 (7.3)	14 (24.6)	0.026
Testicular atrophy, *N* (%)	0 (0)	0 (0)	1.000
Poor wound healing, *N* (%)	0 (0)	1 (1.8)	1.000
Scrotal haematoma, *N* (%)	5 (12.2)	6 (10.5)	1.000
Hernia or hydrocele, *N* (%)	0 (0)	0 (0)	1.000

In terms of postoperative testicular position, the LOMPM group was superior to the LTIO group (lower scrotm: 90.2 vs. 71.9%, *P* = 0.026), and there was a significant difference. Three cases in the LOMPM group were located in the middle scrotum, and 14 cases in the LTIO group were located in the middle scrotum, with a significant difference (*P* = 0.026). One patient in the LOMPM group experienced the testis located in the neck of the scrotum, one patient in the LTIO group experienced the testis located in the neck of the scrotum, and additionally one patient in the LTIO group experienced the testis located in the distal groin. These three patients underwent the reoperation. All patients showed adequate testicular blood flow by color Doppler ultrasound. Testicular atrophy, and inguinal hernia or hydrocele were not found in either group (Table 2).

There was no significant difference in preoperative testicular volume and postoperative testicular volume between the two groups. Postoperative testicular volume in both groups was improved compared with preoperative testicular volume. The testicular growth rate was 1.25 ± 0.55 in LOMPM group, and 1.24 ± 0.51 in LTIO group, but there was no significant difference between the two groups (Table 3).

**Table 3:** Comparison of the testicular volume between the two groups.

	**LOMPM group**	**LTIO group**	***p*-value**
Preoperative testicular volume (ml)	0.23 ± 0.10	0.22 ± 0.08	0.404
Postoperative testicular volume (ml)	0.27 ± 0.13	0.25 ± 0.10	0.549
Growth ratio of testicle	1.25 ± 0.55	1.24 ± 0.51	0.926

## Discussion

NPT accounts for 20–35% of UDT, and NPT patients often have short spermatic cord, which have been a challenge for surgeons (15–17). After the exclusion of non-viable testes, NPT can be classified into intra-abdominal and intracanalicular testes according to the location of the testis (4, 15). Various surgical strategies are currently available to address this problem. High intra-abdominal UDT can be treated with one-stage or two-stage laparoscopic Fowler-Stephens orchiopexy or staged laparoscopic traction orchidopexy (Shehata technique). For low intra-abdominal and intracanalicular testes, many studies suggest that primary laparoscopic orchiopexy can be used (3, 15, 18, 19). In August 2019, our center started using LOMPM for NPT close to the internal ring, and then we compared the surgical outcomes with the historical cohort.

The important factors in judging the success of orchiopexy are the location and viability of the testis (2, 17). Our findings suggestted that the testis after mobilization using the modified Prentiss maneuver has a better scrotal position. After 1 year of follow-up, the testis was located in the low scrotum in 37 patients (90.2%) in the LOMPM group, while the testis was located in the low scrotum in 41 patients (71.9%) in the LTIO group. Yang et al. (5) performed LOMPM on 140 intracanalicular UDT cases, the results of which showed that the low scrotal rate was 89.3%. Elderwy et al. (20) performed LOMPM on 21 peeping testes, and the postoperative follow-up results showed that low scrotal rate was 90.4%. These were basically consistent with our research. In a previous study, Sfoungaris et al. (21) performed inguinal Prentiss orchiopexy on 48 patients, and the gain in cord length was 6–20 mm, with an average of 13 mm. The anatomical principle of this method has been reported by Prentiss et al. (7), and it can offset the spermatic cord medially, and eliminate the angle caused by the path through the internal ring, thereby shortening the distance of testicular descent.

Color Doppler ultrasonography is a reliable tool for measuring testicular volume and testicular blood flow, and is widely used for preoperative examination and postoperative follow-up of NPT (5, 12). We defined testicular atrophy as a loss of more than 50 percent of the post-operative testicular volume relative to preoperative testicular volume (14). None of the patients in this study developed testicular atrophy, and postoperative color Doppler ultrasound showed adequate testicular blood flow, the reasons for which might be that the mobilization of the spermatic cord under laparoscopy is delicate, so that the spermatic cord usually does not be damaged. In addition, we checked the relative position of the spermatic cord to the vas deferens before the testis descended, and used the retractor to expose the spermatic cord after the testis descended to reconfirm that no torsion occurred. The results of Braga et al. (11) and Roy et al. (13) showed that staged laparoscopic Fowler-Stephens procedure for descending the testis through the internal ring could reduce the probability of testicular atrophy. In our present study, the median postoperative testicular volume was 0.27 ± 0.13 ml in the LOMPM group and 0.25 ± 0.10 ml in the LTIO group, with no significant difference. Meanwhile, the median testicular growth rates in the LOMPM group and LTIO group were 1.25 and 1.24, respectively, suggesting that the testicular volume increased postoperatively. These might be due to the fact that the blood flow of the gubernaculum during orchidopexy with sparing spermatic cord is not as important. However, Shiraishi et al. (12) studied the differences in testicular temperature after staged laparoscopic Fowler-Stephens orchiopexy using different descent paths, and the results showed that the temperature of testis descending through the internal ring was more suitable for development. However, whether such differences exist in vessel-intact laparoscopic orchiopexy remains to be further studied.

We used the 1-year postoperative outpatient follow-up results for the above comparison of clinical efficacy, because the results of longer follow-up (testicular position and viability) did not differ from the results at this stage. The results of Elzeneini et al. (17) showed that there was no difference in testicular viability and position at 6 months postoperatively and at 12 or 36 months follow-up. The results of Wu et al. (22) showed that the adverse outcomes of the UDT surgery occurred within 8 months after surgery.

In terms of perioperative complications, 1 patient (1.8%) in the LTIO group had poor wound healing. We considered it to be caused by insufficient expansion of the space between the scrotal skin and dartos, and excessive skin tension after testicular placement. This patient received secondary sutures. Scrotal hematoma is the most common complication of orchiopexy (10). In our study, scrotal hematoma occurred in 5 cases (12.2%) in the LOMPM group, and 6 cases (10.5%) in the LTIO group, all of which were localized hematomas. The cyanosis of scrotal skin resolved spontaneously over time without special intervention. In order to avoid this complication, gentle manipulation and careful hemostasis should be performed during the surgery, and in addition, scrotal compression is also an effective preventive measure (5, 10).

The most critical step of the Prentiss maneuver is the establishment of the channel. In previous studies, this procedure was usually performed using a trocar to enter the abdominal cavity from the scrotum (6, 10). We prefer to use a hemostat for this step. After the hemostatic forceps enters the scrotal incision, we can press the skin with our left hand to sense its advancing direction, and try to fit the pubic bone as much as possible through the transverse abdominal fascia and enter the abdominal cavity. In addition, as opposed to a trocar, the hemostatic forceps can dilate the channel with an open-close operation, ensuring smooth passage of the testis.

In our study, a total of 19 patients (19.4%) had preoperative inguinal hernia or hydrocele. During the intraoperative exploration, 77 cases (78.6%) had patency of the processus vaginalis, which indicated that the patency of the processus vaginalis might not necessarily lead to clinical hernia and hydrocele, and might also explain the pathogenesis of metachronous hernia. Favorito et al. (23) found that the probabilities of the processus vaginalis patency of intra-abdominal and intracanalicular testes were 100% (14/14) and 64.2% (52/81), respectively. Zvizdic et al. (24) suggested that 61.2% of the affected side of UDT near the internal ring had patency of the processus vaginalis patency. Our findings were largely consistent with previous findings (intra-abdominal vs. inguinal, 92.9 vs. 72.9%). The patency of the processus vaginalis is favorable for surgery, and we could relatively easily pull the intracanalicular testes back into the abdominal cavity.

However, we did not suture the peritoneum at the internal inguinal ring, whether the processus vaginalis was patency or closed, and none of the patients developed inguinal hernia and hydrocele after surgery. This approach effectively saves surgery time and reduces the learning curve for surgery. Numerous studies have shown that simply transecting the processus vaginalis does not increase the risk of postoperative inguinal hernia or hydrocele (3, 25). Early animal experiments have shown that the healing process of peritoneal defects is different from that of the skin (26). Instead of crawling from the edge to the center, all defect areas are repaired and fused at the same time (26). This process only takes 5–6 days, and there was no significant difference in the healing time of large-area defects and small-area defects (26). This study provides good support for the need for no suturing of the internal ring peritoneum.

In the treatment of high UDT, the modified Prentiss maneuver has a good clinical effect relative to the descending testis through the internal ring. However, there is still controversy over when to use primary laparoscopic orchiopexy (4). It has been reported that when the testis can reach the contralateral internal ring, primary laparoscopic orchiopexy can be used (3, 5, 6, 16). Bagga et al. (27) confirmed that this was one of the statistically significant factors for predicting primary laparoscopic orchiopexy achievable. However, in the actual surgical procedure, this step is usually performed after extensive retroperitoneal dissociation and mobilization of testicular vessels, at which point the selectivity for staged Fowler-Stephens has been lost. Recent studies have also shown that this criterion cannot guarantee scrotal fixation (15).

At present, the determination of the surgical method in many literatures is based on the distance from the testis to the internal ring. Esposito et al. (18) showed that staged Fowler-Stephens surgery should be performed when the testis is more than 3 cm from the internal ring in the intra-abdominal cavity. Agrawal et al. (19) believed that primary laparoscopic orchiopexy can be used when this distance is <2.5 cm. Bagga et al. (15) indicated that when this distance is <1 cm, vascular intact laparoscopic orchiopexy is the preferred method. This is consistent with our selection criteria and we believe it is appropriate.

In this study, both groups of patients used the dartos pouch technique for reposition of the testis without additional scrotal suture. Hirner et al. (28) suggested that the additional suture in inguinal orchidopexy procedures did not improve the success rate of surgery. A recent meta-analysis showed that orchiopexy performed without transparenchymal suture fixation has an advantage in reducing recurrence rates (29). In our experience, the key to testicular fixation is adequate testicular mobilization and a properly sized dartos pouch. Testicular retraction occurred in 1 patient (2.4%) in the LOMPM group, and 2 patients (3.5%) in the LTIO group. Daboos et al. (3) and Gu et al. (25) treated intracanalicular cryptorchidism with LOMPM and LTIO, respectively, and the probability of testicular retraction was 1.4% (1/70) and 0% (0/137), respectively. This is probably due to the inclusion of low intra-abdominal cryptorchidism in our study. Patients in our study who receive reoperation were all older than 3 years old at the time of initial surgery. The distance from the testis to the scrotum increases linearly with the increase of the age and height (15, 27). Older patients tend to experience greater surgical difficulties in obtaining satisfactory testicular position. In addition, the morphology of spermatic cord is also different, some are small and tense, and some are thick and tortuous. Therefore, when determining the surgical method for high UDT, we should comprehensively considered those factors such as age, the laxity of the spermatic cord, and the distance of the testis from the internal ring.

In addition, there are still some limitations in our study. First, patients in the two groups were included in different periods, which inevitably led to differences in surgical experience. Second, our study did not incorporate measurements of sex hormone levels and lack of follow-up for long-term fertility. Finally, the sample size of this study was small. Therefore, in the future prospective randomized controlled studies with larger sample sizes are needed to confirm our outcomes.

In conclusion, LOMPM is comparable in safety to LTIO, but LOMPM has good post-operative testicular position and is suitable for the treatment of NPT near the internal ring.

## Data Availability Statement

The original contributions presented in the study are included in the article/supplementary material, further inquiries can be directed to the corresponding author.

## Ethics Statement

The studies involving human participants were reviewed and approved by the Ethics Committee of Hunan Children's Hospital. Written informed consent to participate in this study was provided by the participants' legal guardian/next of kin.

## Author Contributions

T-QH and Y-WZ conceived and designed the study. F-YT, YL, and J-JH collected the data. T-QH, ZW, and LT analyzed and interpreted the data. T-QH wrote the manuscript. Y-WZ, JH, Y-FC, and LT provided critical revisions that are important for the intellectual content. T-QH, F-YT, ZW, YL, J-JH, Y-FC, LT, JH, and Y-WZ approved the final version of the manuscript.

## Funding

The Science and Technology Innovation Program of Hunan Province (No. 2020SK50502), National Key Clinical Specialty Construction Project - Pediatric Surgery of Hunan Children's Hospital (XWYF [2022] No. 2).

## Conflict of Interest

The authors declare that the research was conducted in the absence of any commercial or financial relationships that could be construed as a potential conflict of interest.

## Publisher's Note

All claims expressed in this article are solely those of the authors and do not necessarily represent those of their affiliated organizations, or those of the publisher, the editors and the reviewers. Any product that may be evaluated in this article, or claim that may be made by its manufacturer, is not guaranteed or endorsed by the publisher.
